# Parliament and Publications

**Published:** 1911-04-29

**Authors:** 


### PARLIAMENT AND PUBLICATIONS.

### STAMMERING CHILDREN.

The question of the treatment of stammering children is at present receiving the consideration of the Board of Education. Some local authorities make special arrangements for dealing with this defect.

### VACCINATION IN INDIA.

Replying to a question asked by Mr. Chancellor as to whether an investigation of Indian sanitary and vaccination reports had established the facts that during the past forty years what are known as the badly-vaccinated provinces had suffered far less from small-pox than the well-vaccinated provinces, Mr. Montagu said he could not accept the representation of the facts. Vaccination is optional over by far the greater part of British India, including 93 per cent. of the population.

### PUBLIC HEALTH IN SCOTLAND.

The birth rate in Scotland continues to show a marked decline, and in the year 1910 was the lowest which has ever been recorded in official reports. The number of births registered was 124,000, equivalent to a birth rate of 25.16 per thousand, a rate which is 1.20 less than that of the previous year, 2.16 less than the birth rates of the preceding five years, and 3.11 less than the mean of the rates of the preceding ten years. When it is recalled that from 1855 to 1893 the birth rate varied between 30 and 35.6 per thousand, the present rate appears remarkably low. The death rate of the year is also the lowest on record, and 1910 was the first year in which it fell below 15 per thousand. From 1855 to 1880, with the exception of 1856, the national death rate was constantly over 20 per thousand, so that if the statistics relating to births are somewhat disturbing, those relating to deaths reveal a satisfactory improvement in the state of public health. An examination of the death register shows that the year was one without serious epidemic, an outbreak of measles excepted; and the annual death rate from phthisis shows a decrease. The marriage rate, though slightly higher than the previous year, remains low, and is lower than the marriage rate of all previous years since 1855, that of 1909 excepted. The vaccination returns show that the use of the power of exemption from vaccination by reason of the conscientious objection of the parents has been largely taken advantage of, with the result that the proportion of children avoiding vaccination is largely increased. It is estimated that the passing of the Vaccination (Scotland) Act, 1907, has resulted in two years in adding 45,000 unvaccinated children to the population. The number of children reported to have been successfully vaccinated last year, including those certified as insusceptible by reason of previous vaccination, amount to 65.29 per cent. of the total children whose births were registered, and 71.75 per cent. of the children surviving at the time of compulsory vaccination. Children who were not vaccinated by reason of the conscientious objection of their parents numbered last year 22,746, which is the largest number yet recorded. "Fifty-sixth annual report of the Registrar-General in Scotland," Cd. 5617, price 7d.

